# Supplementary figures and images for: A rule-based multiscale model of hepatic stellate cell plasticity: Critical role of the inactivation loop in fibrosis progression
Source: PLoS Comput Biol. 2024 Jul 29;20(7):e1011858. doi: 10.1371/journal.pcbi.1011858 (PMC11309422; doi:10.1371/journal.pcbi.1011858)

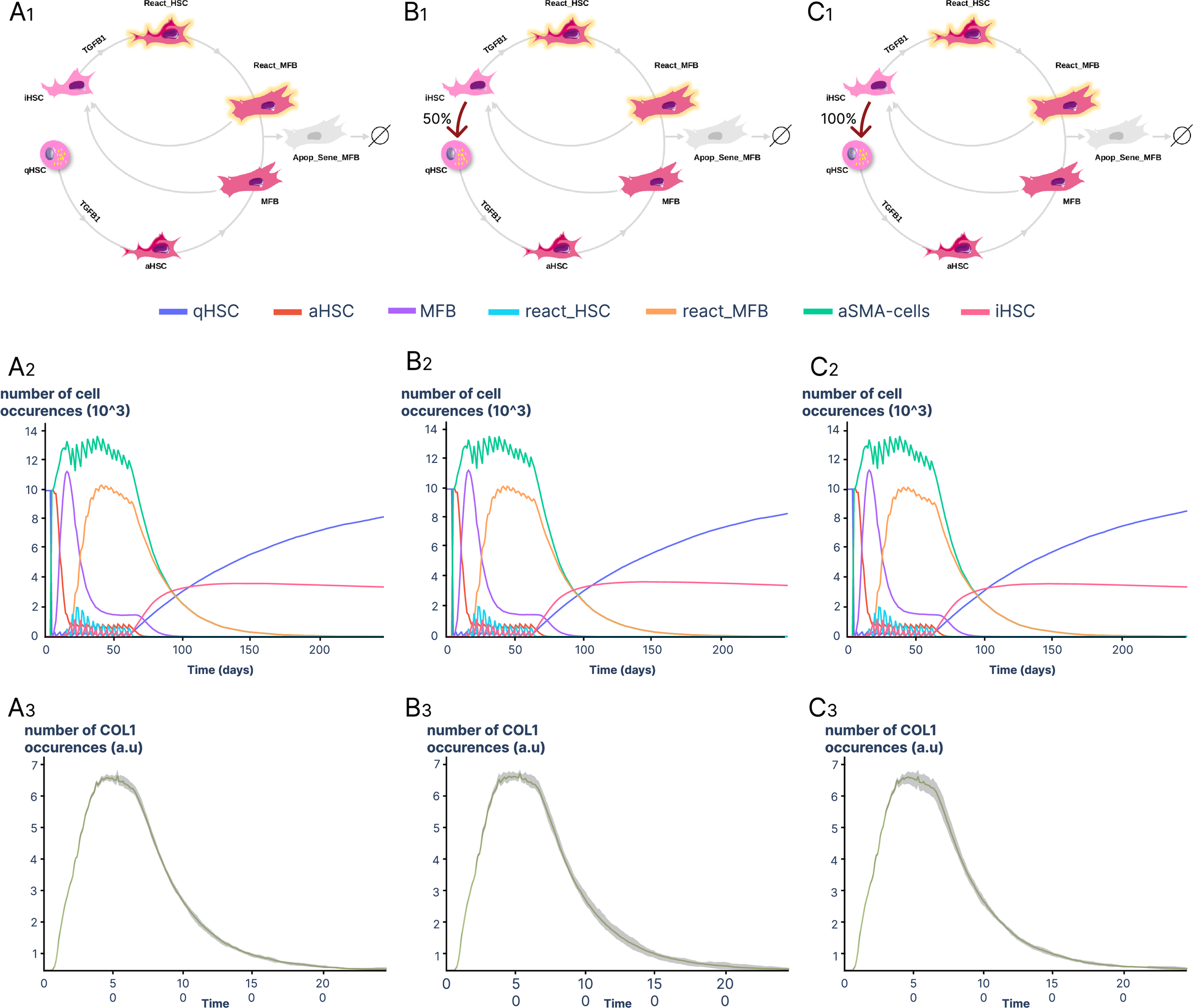

Supplement: S1 Fig — Simulation series were performed using conditions of stimulation from Kisseleva et al. [24]. TGFβ1 parameters were as follows 10,000 molecules per cell, 16 stimulation (twice a week) during 2 months. (A1), models reactMFB-with-inactivation with 0% of the iHSCs reverting into qHSC, (A2) and (A3), models iHSC-reversion-to-qHSC with 50% and 100% of the iHSCs reverting into qHSC, the remaining iHSCs being eliminated. (B1, B2 and B3), variation in the number of cell occurrences for qHSCs, aHSCs, MFBs, iHSCs, react_HSCs, react_MFBs and α-SMA positive cells in the three families of models. The number of α-SMA cells is the sum of the number of aHSCs, MFBs, react_HSCs and react_MFBs. Simulations are expressed as the mean of 10 replicates. (C1, C2 and C3), variation in the number of COL1 occurrence in the three models. Data are expressed as arbitrary unit (a.u) and all 5 replicates are represented. (TIF) [file pcbi.1011858.s001.tif]

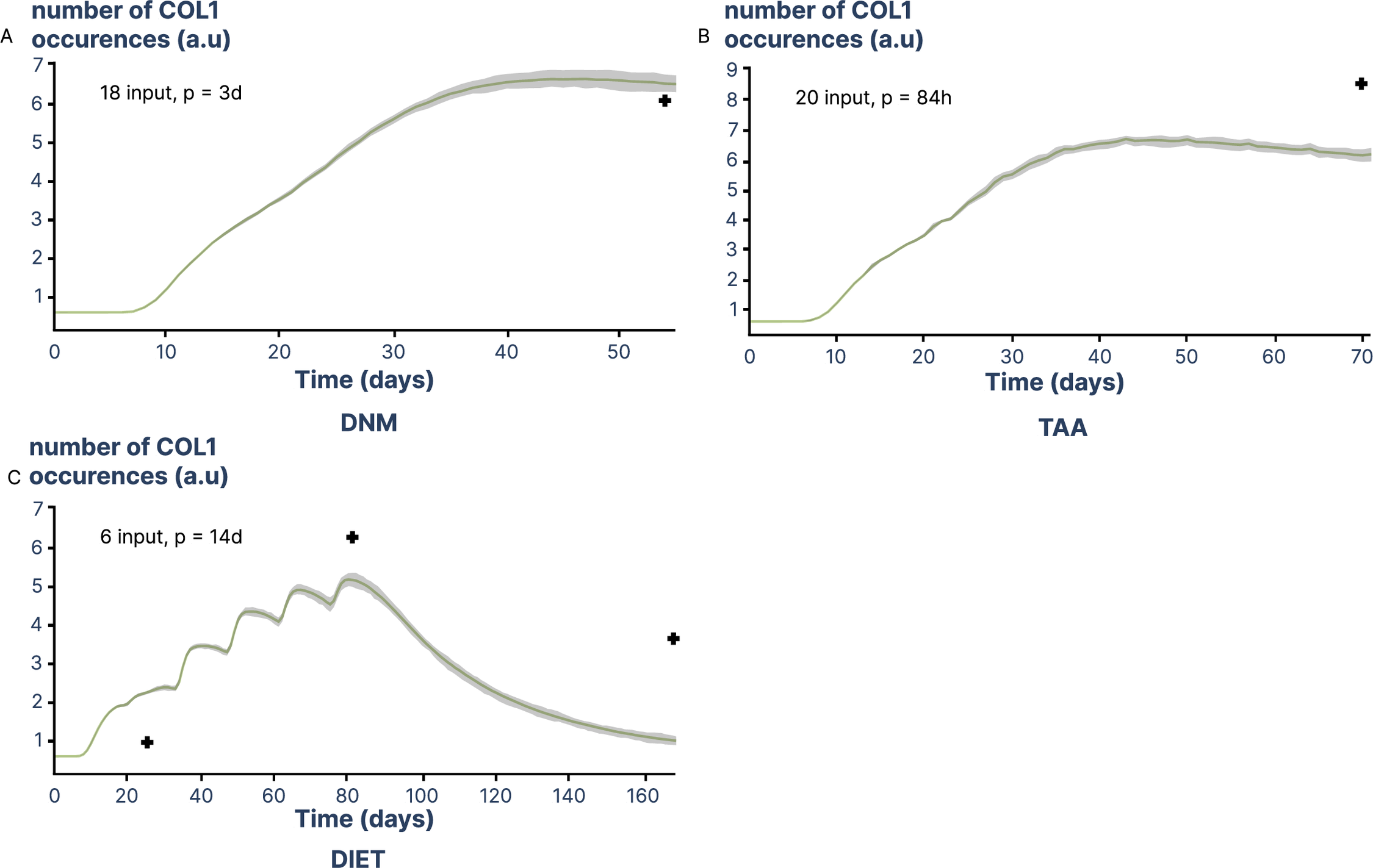

Supplement: S2 Fig — Simulations were carried out using the stimulation protocols described in the different models. (A) dimethyl_nitrosamine model: 3 TGFβ1 stimuli per week for 6 weeks [36], (B) thioacetamide model: 2 simulations with TGFβ1 per week for 10 weeks [37], (C) high-fat model: 6 simulations with TGFβ1 every 14 days for 84 days, adapted to fit Farooq et al. results [55]. Collagen accumulation was plotted and experimental data from [36, 37, 55] are indicated with black cross. (TIF) [file pcbi.1011858.s002.tif]
