# Supplementary material for: A rule-based multiscale model of hepatic stellate cell plasticity: Critical role of the inactivation loop in fibrosis progression
Source: PLoS Comput Biol. 2024 Jul 29;20(7):e1011858. doi: 10.1371/journal.pcbi.1011858 (PMC11309422; doi:10.1371/journal.pcbi.1011858)
Supplement: Attachment — Submitted filename: Cover letter.pdf [file pcbi.1011858.s005.pdf]

irset
Institut de recherche en santé, environnement et travail

Instituts thématiques Inserm
Institut national de la santé et de la recherche médicale

Rennes, May, 2024

To the editor in chief

We thank you very much for giving us the opportunity to submit a revised version of our manuscript entitled "A rule-based multiscale model of hepatic stellate cell plasticity: critical role of the inactivation loop in fibrosis progression", and we thank the reviewers, who helped us to improve the manuscript.

We have carefully considered the points raised by the three reviewers and we have addressed all concerns namely by adding detailed explanation of the methodology including parameter estimation.

We hope that our revised manuscript, which we believe brings important new insights in dynamics of liver fibrosis, will now be considered suitable for publication in PLOS Computational Biology.

The data reported in this manuscript have never been published and are not submitted for publication elsewhere. All the authors are aware of, and agree to, the contents of the manuscript. The authors declare that they have no conflict of interest.

We thank you for considering our submission.

Yours sincerely,

Nathalie THERET, Ph.D.

#REVIEWER 1

I am not qualified to assess the biological subject matter (I do not know much about livers) so I will confine my comments to the methodology which is sound and a crisp illustration of two mechanisms available in the Kappa language. Most of my comments relate to clarity of exposition which the authors may take or leave at their discretion. One criticism, about parameter estimation, is more substantive, and there is one error about a citation in the text.

Reviewer: The authors present three models, HSC_dynamics_model, HSC_dynamics_model_iHSC_reversion, HSC_dynamics_model_without_reactMFB_inactivation as named in the source code repository (they are called something different in the text -- we may want better naming consistency).

Authors: We agree with the reviewer and the model family names have been renamed in the GitHub repository for consistency.

Reviewer: It might be a good idea to explicitly describe their relationship in the main text. Rather than saying "we developed three models ... this model has 75 rules, that model has 76 rules and the other model has 77 rules" (page 4, line 104 onwards), to say something along the lines of, "to get 'reversion' from 'inactivation' we change the rate of one rule and add one extra rule". This would give a better sense of how far in the landscape of models they are from one another and that the changes are really very local. To be fair, Figure 1 does convey this sense but the text could give a different impression (this impression might be an artefact of silly journal rules that say for review purposes, the figures should be as far away as possible from where they belong)

Authors: We apologize for the lack of explanation of the links between the different models in the body of the text. To take this into account, we have modified the text on page 4 to clarify the relationships between the three model families.

Reviewer: The introduction to the Kappa syntax explains about bonds. However, I think this misses the point somewhat. We could imagine a model without tokens where agents A and B had to explicitly bond to one another. This would be computationally expensive so instead of using a bond, we can say that an agent A bonds to some B but we do not care which B. It is enough simply track the bound state and the number of available Bs. No bonds are used in these models and they are not necessary. Using examples (eqs 2-3) that involve explicit bond formation obscures what I believe is a central point that you are trying to make. Having explained about the use of tokens to avoid the expense of explicit bonds, then explain counters and what, in general terms, they are used for here. I suggest rewriting this section to make it more clearly illustrate these mechanisms as used in these models.

Authors: We agree that the explanation concerning tokens and counters is not very clear and the section has been rewritten in the revised version of the manuscript on page 14.

Reviewer: In the models there is an idiom used of a counter called "intermediate_step" together with another called "control_counter". Whilst biologically irrelevant it is necessary for the functioning of the model. Perhaps it would be useful to explain why this is necessary

as it would be puzzling for someone unfamiliar with this idiom who tries to read the model. A better solution would be to modify the Kappa language so that this is not necessary.

Authors: While the use of the so-called "intermediate_step" counter is biologically relevant based on discrete experimental data regarding the HSC activation process, we agree that the "control_counter" we introduced to compensate the Kappa syntax, requires further explanation. Modifying the Kappa syntax is outside the scope of this study, but current developments of the Kappa syntax are addressing this issue. In line with the reviewer's comment, we have modified the text on page 14 and 15 to detail the use of "control_counter".

Reviewer: Some model parameters come from the literature, some are sensibly calculated and some are fitted. I found the discussion of how they were fitted to be lacking. "22 parameters were estimated from biological data ... numerous simulations before finding the correct set of parameters". This is quite a strong claim for such a large number of free parameters especially given the acknowledged "interdependence between the parameters". How do you know these are "correct" as opposed to one of many possible choices that are consistent with the biological data? The precise method used is left rather vague. I think we need more detail here.

Authors: Parameter estimation in our models was particularly tricky in this study and we agree that the method needs to be further described. In brief, we used a block-based parameter estimation method, i.e. a set of parameters for each subsystem (block) was estimated sequentially. Each set was defined in terms of a specific biological process such as cell proliferation, cell inactivation, collagen production and degradation. Block estimation reduced the impact of interdependence and enabled us to find a set of parameters corresponding to biological observations. As the evaluation of collagen was the experimental model best described, we first calibrated the set of parameters related to collagen-producing cells. Next, we identified the set of parameters related to react-MFB inactivation which strongly affects model dynamics. The third set of parameters focused on iHSC and qHSC dynamics. The final set of parameters focused on collagen itself, encompassing its production and degradation. Following this approach, the estimation of the first set of parameters excluded the family of models called reactMFB-wo-inactivation, as shown in Figure 2A. Finally, we performed a global verification to determine whether all the parameters matched the experimental observations (visual inspection). The method is now detailed in the revised version of the manuscript (page 18).

Reviewer: In the discussion of parametrisation, several parameters are swept. This is fine, but the language used is of the form, "we developed models by varying the parameters", suggesting that changing parameters means different models. That is, there are not three but an unbounded number of models! This is a subtle point and might seem minor, and we could talk about parametrised families of models so really what we have here is three families. This is a perennial problem in much of the literature where the word "model" is used to mean many different but related things. I think the paper would benefit from setting a good example by making the language more precise.

Authors: We agree with the reviewer that the use of "models" and "model families" should be standardized in this field of research. We have modified the text accordingly throughout the revised version of the manuscript.

Reviewer: A rule-based model using counters by Waites et al is described as unpublished and cited in a footnote. That model (with a slightly incorrect URL, LaTeX appears to have swallowed the ~ character) is in fact the supporting material for this published paper https://royalsocietypublishing.org/doi/10.1098/rsta.2021.0307.

Authors: We sincerely apologize for this error and the reference was corrected in the revised version of the manuscript (page 11).

As additional comment, the reviewer questioned the availability of data used for parameterization**.**
Author: We added the python script used for launching the simulations on a git-hub mentioned on page 21

#REVIEWER 2

The authors present a rule-based model of liver fibrosis focused on the role of hepatic stellate cells (HSC). The model presented in this manuscript builds upon prior agent- and equation-based models of liver fibrosis by including HSC explicitly. The behavior of these cells is fairly granular and includes various states of activation. This model, which was partially validated against data from mice undergoing liver fibrosis secondary to CCl4 administration, suggests a role for TGF-β1-related parameters. Furthermore, the model was used to predict features of chronic liver disease in humans.

Reviewer: While the paper is generally well-written and the modeling work is sound, one key issue needs to be addressed by the authors. This relates to the human validation data (Fig. 6 and related studies). Specifically, this concerns the data on iHSC gene expression. In this analysis, the authors show a ~1.4-fold elevation in iHSC gene expression, with a p value of 0.097. In general, gene expression fold changes below 2-fold, and with p value above 0.05, are not considered relevant. The addition of several genes pushed the fold change in expression to ~1.6 (still less that twofold, albeit with $p < 0.05$). Thus, these data would be interpreted most conservatively as not showing any major difference, and thus the statement that "….these observations suggest the presence of an increased number of iHSCs during the progression of liver fibrosis, validating the predictions of (the) model," and similar statements in the Discussion, should be tempered to reflect these relatively weak data. I would suggest noting that these data do not disprove model predictions, but that more data are needed to fully support that hypothesis.

Author: Concerning the human validation data, we understand the reviewer's question about the value of the normalized enrichment scores (NES) calculated by the GSEA method. The reviewer is quite right that for the expression of a single gene, an increase greater than two-fold with a p-value lower than 0.05 is generally considered relevant. However, here, we are not considering the expression of a single gene, but the simultaneous enrichment of a set of genes (n=39 in Fig6A or 49 in Fig6B), forming a signature. Consequently, the NES value is not equivalent to a fold-change value. This being the case, we agree with the reviewer that the nominal p-values remain at the limit of significance but the trajectory of score improvement between the two trials (Fig6A and Fig6B) unquestionably reinforces our interpretation. To take into account the reviewer's remark, we have tempered our interpretation of these GSEA analyses both in the Result and in the Discussion sections. The text is modified accordingly (Pages 9 and 13).

#REVIEWER 3

****Summary:
The authors developed models to explain the role of hepatic stellate cells (HSC) in the progression and reversal of fibrosis including detailed mechanism involved. Based on information about the processes, they built 3 “candidate” models based on the current biological knowledge using Kappa rules (a graph rewriting language) to decipher the importance of apoptosis, trans-differentiation and reactivation of HSC cells in the fibrosis reversal/progression processes. Based on simulation results, model "reactMFB-with-inactivation" was selected. Model selection was based on comparisons of model simulations and data both qualitative and quantitative, showing that reactivation loops of inactivated HSCs is particularly relevant during sustained fibrosis. However, the methodology used for the joint estimation of parameters and how this affects the simulations and model selection is not completely clear. And the complexity of the model does not make it easy to decipher the effect of these empirical adjustments on the result of the simulations to validate/select the model. A more detailed explanation of the methodology used could help clarify these points, as well as the incorporation of statistical analysis for model selection. Finally, the authors describe in detail each of the processes incorporated in the model, contrasting it with reported data, highlighting the role of the HSCs reactivation process and the accumulation of inactivated HSCs as possible markers of fibrosis progression.

Reviewer: Introduction. The authors could include a brief explanation of why the Kappa language was used to model this system.

Authors: The choice of the Kappa language was explained in the discussion section. We have now included this explanation in the introduction to the revised manuscript on page 3.

Reviewer: Results, lines 138 – 160. Description of Figure 2 helps to understand the deduction of why the reactMFB-with-inactivation model is more appropriate than the reactMFB-wo-inactivation model, however the graphical description could be improved. For example, clearly showing the point at 8 weeks where a difference in the behavior of alpha-SMA expressing cells and COL-1 accumulation dynamics is observed across the models.

Authors: As requested by the reviewer, we have modified Figure 2 by adding red circles to show experimental values at 8 weeks. The legend of figure 2 has been modified accordingly (page 6).

Reviewer: Results, lines 138 – 168. Although the deduction of why the react_MFB inactivation loop understandable due to reported data, a comparison of averages is not enough to say that it is "essential". Could the authors provide confidence intervals from simulations and show that the model without loop (0% of react_MFB inactivation) presents significant differences to the model with the loop (react_MFB inactivation of 5%)? A statistical test comparing simulated versus reported results at the times of interest would help support these findings.

Authors: In line with this remark, we have added standard deviation values for simulation points at 8 weeks that exclude the models without loop (0% of react_MFB inactivation). The text has been modified on pages 5-6 in the revised version of the manuscript.
A statistical test comparing the simulated results with the results reported at this time point is not necessary since the models without loop (0% of react_MFB inactivation) showed a decrease in α-SMA-producing cells whereas an increase of this parameter is observed in biological experiments.

Reviewer: In Figure captions review concepts of "Data" vs Simulation results. For example, in figure 3 it is understood from the text that it corresponds to simulations, no data.

Authors: We apologize for this misuse of language and the text of the figure legends has been corrected.

Reviewer: Results, lines 163 – 165. Could the authors explain why the results of the iHSC-reversion-to-qHSC model are practically the same as the results of the reactMFB-with-inactivation model? The choice of the reactMFB-with-inactivation model based on this comparison is supported by these results, but it is not explained why. Also, could the parameter estimation methodology affect this decision (because of practical identifiability problems)? It possible to justify the choice of the model using a selection criterion such as AIC, BIC, AICc, or others, using data and the simulations results?

Authors: We agree that the results of the iHSC-reversion-to-qHSC model are virtually the same as those of the reactMFB-with-inactivation model. As indicated in the discussion section, this may be due to the slow dynamics of iHSC elimination and quiescent cell turnover, which limits the impact of reversion. We have discarded this model, which was developed on the hypothesis of iHSC reversion but without any supporting experimental observations. To take of this comment into account, we have modified the text on page 6.

Reviewer: Results, lines 205 – 231. It would be interesting to know the prediction of the other two discarded models with respect to the data used to validate the model and compare the results.

Authors: As no set of parameters corresponding to biological observations was found for the reactMFB-wo-inactivation models, no predictions were possible. With regard to the exploration of iHSC-reversion-to-qHSC models, we have chosen not to explore these models in depth due to the lack of supporting experimental observations.

Reviewer: - Methods, lines 591-595. The authors state that 22 parameters were estimated from biological observations "running hundreds of simulations", whose values are interdependent. They also indicate that "some parameters were estimated empirically by groups (…), leading to numerous simulations before finding the correct set of parameters". The following questions remainun. answered:
1. How do authors determine which is the correct set of parameters? Was an objective function used for model calibration or just visual inspection of simulations?
2. How were the groups of parameters stablished for the "empirical" estimation?
3. How the interdependency between parameters was considered to determine the groups of parameters that were "empirically estimated"?

- Methods. pages 16-18. Given the interdependence of parameter values that the authors established, it is difficult to know whether the data used for calibration are sufficient to estimate 22 model parameters. Could the authors clarify which parameters are those that present a high correlation, or interdependence and provide confidence intervals for each of the estimated parameters?
- Methods. pages 16-18. Model calibration and rules for parameters are described by the authors, but it is not clear how they calibrated the model using the reference data, that is, whether an objective function existed to minimize the difference between predictions and data, and how the values were simultaneously estimated (considering the different established rules and the interconnection between them can affect indirectly the estimation of parameters involved in other mechanisms).

Authors: We agree with the reviewer regarding the lack of explanation of the method we used to calibrate the model. In response to reviewer 1, we have detailed the method in the revised version of the manuscript as follows: "*To calibrate the models, we used a block-based parameter estimation method, i.e. a set of parameters for each subsystem (block) was estimated sequentially. Each set was defined in terms of a specific biological process such as cell proliferation, cell inactivation, collagen production and degradation. Block estimation reduced the impact of interdependence and enabled us to find a set of parameters corresponding to biological observations. As the evaluation of collagen was the experimental model best described , we first calibrated the set of parameters related to collagen-producing cells. Next, we identified the set of parameters related to react-MFB inactivation which strongly affects model dynamics. The third set of parameters focused on iHSC and qHSC dynamics. The final set of parameters focused on collagen itself, encompassing its production and degradation. Following this approach, the estimation of the first set of parameters excluded the family of models called reactMFB-wo-inactivation, as shown in Figure 2A. Finally, we performed a global verification to determine whether all the parameters matched the experimental observations (visual inspection)*".
The text mas modified on page 18.

Reviewer: - Discussion- Conclusion. In the abstract it is mentioned that understanding the dynamics of HSC will provide insights needed to develop new therapeutic approaches. Could the authors discuss how the new model could contribute to developing new therapeutic approaches? Could the developed model help find the critical points (and critical parameters) where reversal vs. sustained fibrosis occurs?

Authors: In the abstract, we postulate that understanding HSC dynamics will offer new opportunities, and our model identifies iHSC accumulation as a novel marker of fibrosis progression. The use of such markers can improve the monitoring of patients, leading to new, more personalized therapeutic approaches.
Yes, our models can identify the critical threshold of iHSC above which the tissue can be repaired and below which there is a risk of disease worsening in the mouse model. However, complete recalibration would be required to extrapolate the model to fit human disease dynamics.
